# Artificial biosynthesis of phenylpropanoic acids in a tyrosine overproducing *Escherichia coli* strain

**DOI:** 10.1186/1475-2859-11-153

**Published:** 2012-12-03

**Authors:** Sun-Young Kang, Oksik Choi, Jae Kyung Lee, Bang Yeon Hwang, Tai-Boong Uhm, Young-Soo Hong

**Affiliations:** 1 Chemical Biology Research Center, Korea Research Institute of Bioscience and Biotechnology, 30 Yeongudanji-ro, Ochang-eup, Chungbuk 363-883, Republic of Korea; 2 Department of Pharmacy Graduate School, Chungbuk National University, Cheongju, 361-763, Republic of Korea; 3 Biological Sciences, Chonbuk National University, Jeollabuk-do, 561-756, Republic of Korea

## Abstract

**Background:**

The phenylpropanoid metabolites are an extremely diverse group of natural products biosynthesized by plants, fungi, and bacteria. Although these compounds are widely used in human health care and nutrition services, their availability is limited by regional variations, and isolation of single compounds from plants is often difficult. Recent advances in synthetic biology and metabolic engineering have enabled artificial production of plant secondary metabolites in microorganisms.

**Results:**

We develop an *Escherichia coli* system containing an artificial biosynthetic pathway that yields phenylpropanoic acids, such as 4-coumaric acid, caffeic acid, and ferulic acid, from simple carbon sources. These artificial biosynthetic pathways contained a codon-optimized *tal* gene that improved the productivity of 4-coumaric acid and ferulic acid, but not caffeic acid in a minimal salt medium. These heterologous pathways extended in *E. coli* that had biosynthesis machinery overproducing tyrosine. Finally, the titers of 4-coumaric acid, caffeic acid, and ferulic acid reached 974 mg/L, 150 mg/L, and 196 mg/L, respectively, in shake flasks after 36-hour cultivation.

**Conclusions:**

We achieved one gram per liter scale production of 4-coumaric acid. In addition, maximum titers of 150 mg/L of caffeic acid and 196 mg/L of ferulic acid were achieved. Phenylpropanoic acids, such as 4-coumaric acid, caffeic acid, and ferulic acid, have a great potential for pharmaceutical applications and food ingredients. This work forms a basis for further improvement in production and opens the possibility of microbial synthesis of more complex plant secondary metabolites derived from phenylpropanoic acids.

## Background

Phenylpropanoic acids, such as 4-coumaric acid, caffeic acid, and ferulic acid, are natural phenolic compounds derived from the phenylpropanoid pathway [1]. Phenylpropanoic acids have attracted increasing attention for their various pharmaceutical properties as well as a valuable monomer for the production of liquid crystal polymers, which can be used for electronic applications [2-4]. Although these compounds are widely used in human health care and industrial material, at present they are mainly obtained by extraction from plants, and extraction yields are low because most of these metabolites accumulate at low levels in plant cells. Furthermore, their isolation from plant material, especially as pure compounds, remains a challenge. Nowadays, large-scale production of plant metabolites or precursors via microbial approaches provides a promising alternative to chemical synthesis and extraction from plant sources [5-7].

**Figure 1:**
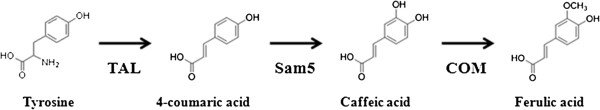
**Proposed ferulic acid biosynthetic pathway starting with L-tyrosine.** TAL, tyrosine ammonia lyase; Sam5, 4-coumarate hydroxylase; COM, caffeic acid methyltransferase.

4-Coumaric acid, caffeic acid and ferulic acid are aromatic compounds containing a phenyl ring with a C3 side chain. These phenylpropanoic acids are the pivotal intermediates of the plant phenylpropanoid pathway starting from the deamination of tyrosine. Tyrosine ammonia-lyase (TAL) catalyzes nonoxidative deamination of the primary amino acid tyrosine to 4-coumaric acid (Figure 1). 4-Coumaric acid is converted into caffeic acid by a hydroxylation step at the 3-position of the benzyl ring. The 4-coumarate 3-hydroxylase (C3H) in plants is composed mainly of cytochrome P450 dependent monooxygenases. The purification and characterization of this enzyme is quite challenging because of its instability and membrane-bound property [8]. Recently, a microbial C3H (Sam5) from the actinomycete *Saccharothrix espanaensis* was reported and its function in *Escherichia coli* was characterized [9]. 4-Coumaric acid is easily converted into caffeic acid by *E. coli* containing the Sam5 expression vector, which was also demonstrated in our previous report [10]. Ferulic acid is biosynthesized from caffeic acid by the enzyme caffeate *O*-methyltransferase [10,11].

Like many other plant secondary metabolites, the production yields of phenylpropanoic acids, notably caffeic acid and ferulic acid, are still low because of the complex and strict regulation of their biosynthesis in plant cells [12,13]. Chemical and enzymatic methods have also been used to produce those phenylpropanoic acid derivatives. Recently, there has been increasing interest in microbial production of phenylpropanoic acid derivatives by reconstructing their biosynthetic pathways in microorganisms. Sachan et al. reported the co-production of caffeic acid and *p*-hydroxybenzoic acid in *Streptomyces caeruleus* by feeding 4-coumaric acid [14]. The conversion of tyrosine to caffeic acid (50.2 mg/L) in *E. coli* was achieved by the co-expression of the enzymes encoded by the TAL from *Rhodobacter* and *E. coli* native 4-hydroxyphenylacetate 3-hydroxylase (4HPA3H) [15]. We also reported the production of ferulic acid in *E. coli* by the sequential co-expression of the enzymes encoded by the *sam5* and *tal* genes from *S. espanaensis* and an *O*-methyltransferase (COM) gene from *Arabidopsis thaliana*[10]*.*

**Figure 2:**
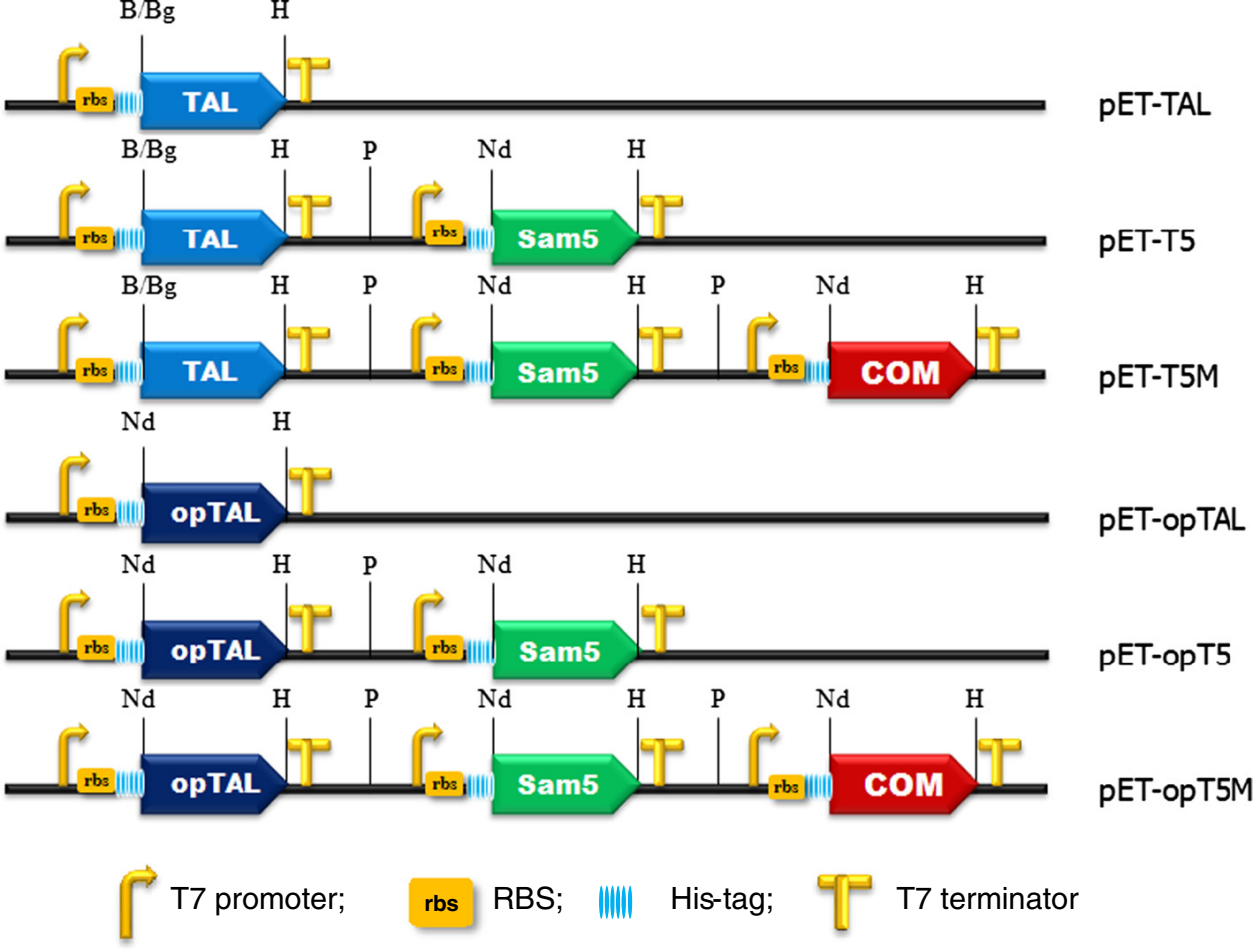
**Organization of the artificial gene clusters used for production of each phenylpropanoic acid in *E. coli*.** Three heterologous enzymes must be expressed in *E. coli* to mediate the synthesis of ferulic acid from tyrosine. All constructs contained the T7 promoter and the RBS in front of each gene and the T7 terminator located in the rear of each gene. opTAL is a codon-optimized TAL enzyme. B, *Bam*HI; Bg, *Bgl*II; H, *Hind*III; P, *Pst*I; Nd, *Nde*I.

Although previous studies have already made significant gains in demonstrating the feasibility of microbial production in *E. coli*, the biosynthetic efficiency is greatly limited by the heterologous gene expression and/or host cellular primary precursor productivities. Because tyrosine serves as the main precursor for phenylpropanoids, strains exhibiting an enhanced capacity for its synthesis provide a natural platform for exploring the potential of microbial phenylpropanoid production from glucose [16,17]. In this study, we made an *E. coli* strain capable of high-level tyrosine production containing feedback-inhibition *aroG* and *tyrA* genes by modifying a previously reported expression system [18,19]. The serial artificial biosynthetic gene expression sets encoding TAL, TAL & Sam5, and TAL & Sam5 & COM, graft into the *E. coli* that is overproducing tyrosine (Figure 2). This method led to the efficient production of serial phenylpropanoic acids, 4-coumaric acid, caffeic acid and ferulic acid. The other three artificial expression sets containing the codon-optimized *tal* gene tested the improvement gene expression effect with respect to phenylpropanoic acid production. In these systems, engineered *E. coli* cultured in a growth medium, even through a simple fermentation in minimal media without precursor supplementation, produced the plant-specific 4-coumaric acid, caffeic acid and ferulic acid to yield 974 mg/L, 150 mg/L, and 196 mg/L, respectively. This report describes a convenient and efficient system for production of 4-coumaric acid, caffeic acid and ferulic acid by microorganisms. There is an obvious economic incentive to develop strains capable of converting cheaper feedstocks to high value compounds.

## Results and discussion

### Comparison of *in vivo* activities of TAL and codon-optimized TAL (opTAL)

TALs identified from various sources can catalyze the direct formation of 4-coumaric acid from tyrosine. We have already succeeded in synthesizing 4-coumaric acid in *E. coli* from a simple medium without the addition of tyrosine using TAL from *S. espanaensis*[10]. The production of ferulic acid in *E. coli* was also achieved by the co-expression of the enzymes encoded by the artificial biosynthetic genes, *tal*, *sam5*, and *com*. However, the production yield of ferulic acid using our previous strategy was less than 10 mg/L [10]. Normally, actinomycete genes are often difficult to express in *E. coli*. They might contain codons and alterations of mRNA structural elements that are rarely used in *E. coli*[20,21]. In practice with the pET system and other high-level *E. coli* expression systems, the presence of a small number of rare codons often does not severely depress target protein synthesis. To address this possibility, we tried to make the ‘opTAL’ protein using the codon-optimized program for overcoming the codon bias of the actinomycete gene for enhanced protein expression in *E. coli*.

**Figure 3:**
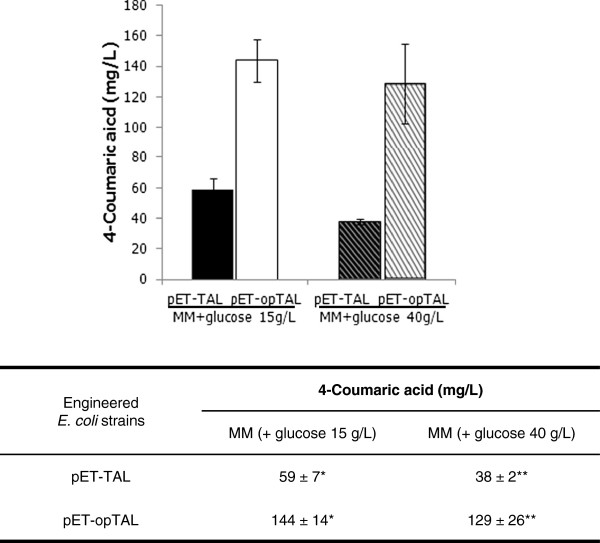
**Comparison of TAL activity.** Concentrations of 4-coumaric acid in strains with pET-TAL or pET-opTAL cultures in an M9 medium containing glucose 15 g/L (Black bar and white bar) or 40 g/L (bo1d diagonal bar and thin diagonal bar). Each cultivation was done at least in triplicate, and the standard deviations are shown. Error bars indicate standard errors of the means. The codon-optimized TAL activity and glucose effect were compared by single-factor ANOVA (*P* < 0.05) using Microsoft Excel. Different stars codes (*, **) indicate significant differences (*, *P* = 9.44E-5; **, *P* = 1.62E-4).

The genes encoding the two TALs were cloned and expressed in *E. coli* using the plasmids pET-TAL and pET-opTAL (Figure 2). The transformant cells were found in a large amount of TAL in both the insoluble and soluble fractions, when cultured at 37°C (Additional file 1: Figure S1). The transformant cells were cultured at 26°C to avoid the formation of inclusion bodies from the proteins. Even by culturing at 26°C, a considerable amount of the 56 kDa TAL was still recovered in the insoluble fraction (data not shown). To evaluate the performance of the two TALs in *E. coli*, we assessed 4-coumaric acid production by culturing the recombinant strains in M9 minimal media (with 15 g/L or 40 g/L glucose) supplemented with 1 mM IPTG. Analysis of the product after 36 hours showed that the production of the 4-coumaric acid reached up to 144 ± 14 mg/L from the pET-opTAL strain in a 15 g/L glucose medium, which was 244% higher than the production from the parental strain, pET-TAL (59 ± 7 mg/L) (Figure 3). The codon-optimized TAL showed a marked effect in the native tyrosine pathway of *E. coli*. On the other hand, 4-coumaric acid synthesis in a 40 g/L glucose medium was 38 ± 2 mg/L (pET-TAL) and 129 ± 26 mg/L (pET-opTAL). However, there was not a statistical significantly difference between the titers for the 15 g/L and 40 g/L glucose samples in media (α = 0.05) (Additional file 1: Table S1). Therefore, the following experiments were performed with an M9 minimal medium with 15 g/L glucose.

### Effects of codon-optimized TAL on caffeic acid and ferulic acid production

The functionality of this codon-optimized TAL (opTAL) with Sam5 and Sam5 plus COM within *E. coli* was evaluated by monitoring the accumulation of caffeic acid and ferulic acid in the culture supernatant. We have already succeeded in synthesizing ferulic acid using the artificial biosynthetic pathway containing the parental TAL [10]. Thus, to obtain caffeic acid from a simple medium without the addition of tyrosine, we made an additional expression vector, pET-T5. In addition, we constructed the same sequential expression vectors, pET-opT5 and -opT5M, containing the codon-optimized *tal* gene. The sequential expression vectors were also equipped with their own T7 promoter, ribosome binding sites (RBS) and terminator to facilitate strong and inducible expression within the *E. coli*, as in the parental vectors (Figure 2).

To assess the caffeic acid biosynthesis, recombinant *E. coli* strains with pET-T5 or pET-opT5 were cultured in M9 minimal media supplemented with 15g/L glucose as the sole carbon source (Table 1). Upon IPTG induction, the caffeic acid secreted in the culture media was analyzed using high-performance liquid chromatography (HPLC) after 36 hours. In this case, the synthesis of caffeic acid in *E. coli* cells with pET-T5 reached 42 ± 19 mg/L and with pET-opT5 reached 14 ± 2 mg/L. Contrary to our expectations, the caffeic acid synthesis in *E. coli* cells with pET-opT5 did not improve. Rather, the titer showed a pattern of decrease, even if the difference was not significant (*P* = 0.066). The low caffeic acid productivity in comparison with the previous 4-coumaric acid was perhaps due to poor expression of Sam5 proteins coupled with high expression of codon-optimized TAL protein. To address this possibility, the engineered strains were tested for protein expression via SDS–PAGE analysis (Additional file 1: Figure S2). However, a considerable amount of the 59 kDa Sam5 protein was detected as having opTAL protein (56 kDa), and the expression level of the parental TAL protein (different His-tagged sequences; 57.4 kDa) showed a tendency to overlap with the Sam5 protein itself (Additional file 1: Figure S2). Thus, the low production of caffeic acids in the opTAL-containing pathway (pET-opT5) was not a result merely of poor protein expression, but may instead be related to inherent deficiencies in enzyme activity. We suspected that Sam5 activity may present a major bottleneck in these strains and culture conditions.

**Table 1:** **Production of caffeic acid and ferulic acid by *E. coli *strains with pET-T5 and pET-opT5 or pET-T5M and pET-opT5M**

**Engineered *E. coli *strains**	**4-Coumaric acid (mg/L)**	**Caffeic acid (mg/L)**	**Ferulic acid (mg/L)**
pET-T5	ND	**42 ± 19**^**a**^	*-*
pET-opT5	26 ± 8	**14 ± 2**^**a**^	*-*
pET-T5M	ND	ND	**28 ± 10**^**b**^
pET-opT5M	ND	ND	**73 ± 15**^**b**^

The engineered strains are cultured in an M9 medium containing glucose 15 g/L. Each batch cultivation was done at least in triplicate, and the standard deviations are shown. Different letter codes (a and b) indicate significant differences (a, *P* = 0.0664; b, *P* = 0.00226). The code ‘ND’ indicated that something was not detected in our HPLC analysis.

On the other hand, ferulic acid synthesis in *E. coli* cells harboring pET-T5M and pET-opT5M was 28 ± 10 mg/L and 73 ± 15 mg/L, respectively, which was a 260% improvement in the codon-optimized TAL construct. An unexpected improvement of the ferulic acid titer compared with the caffeic acid titer in each codon-optimized TAL construct was observed (14 mg/L up to 73 mg/L). The reason for this titer improvement is still unknown, but it is possible that the metabolic flow to ferulic acid may be alleviating any restraint during accumulation of caffeic acid in the cell.

### Construction of tyrosine overproducer and improvement of 4-coumaric acid production in the tyrosine overproducing strain

In order to investigate the effect of intracellular tyrosine concentration on phenylpropanoid biosynthesis, the production levels of 4-coumaric acid, caffeic acid and ferulic acid from the tyrosine overproducing strain were evaluated. The biosynthesis of aromatic amino acids like tyrosine was very tightly regulated by the concentration of the final products. We first generated an *E. coli* strain that disrupted the *tyrR* gene, the product of which represses the expression genes involved in aromatic amino-acid biosynthesis [22]. In addition, the feedback-inhibition-resistant (fbr) 3-deoxy-*d*-arabino-heptulosonate-7-phosphate (DAHP) synthase (AroG^fbr^) and chorismate mutase/prephenate dehydrogenase (TyrA^fbr^) were overexpressed as previously reported by Lütke-Eversloh and Stephanopoulos [18]. Thus, the tyrosine over-producing *E. coli* strain contains an in-frame deletion in *tyrR* and a plasmid (pAD-AG) expressing the *aroG*^*fbr*^ and *tyrA*^*fbr*^. The engineered *E. coli* cells produced tyrosine in the M9 modified medium at a yield of ~400 mg/L, when glucose was used as the carbon source (data not shown). By contrast, the *E. coli* wild-type cells produced low quantities of tyrosine in culture, which was not detectable in our experimental conditions. The tyrosine production yield was comparable to the *E. coli* T1 strain (346 mg/L) as previously reported by Lütke-Eversloh and Stephanopoulos [18]. The *E. coli* T1 strain was made by introducing the constitutive promoter P_Ltet−O1_ for the expression of the *aroG*^*fbr*^ and *tyrA*^*fbr*^ genes in the low-copy-number (~5 copies/cell) plasmid pCL1920, but our two fbr genes were cloned on the inducible T7 promoter of the medium-copy-number (20–30 copies/cell) plasmid pACYCDuet-1.

**Figure 4:**
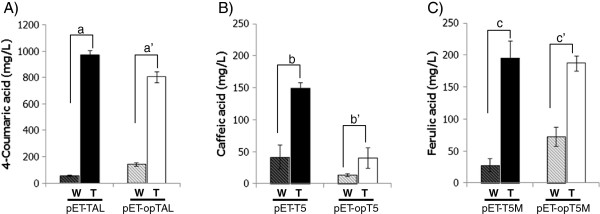
**Comparison of 4-coumaric acid (A), caffeic acid (B) and ferulic acid (C) between wild-type and engineered tyrosine overproducing *E. coli *strains.** (**A**) Production of 4-coumaric acid with pET-TAL and pET-opTAL; (**B**) Production of caffeic acid with pET-T5 and pET-opT5; (**C**) Production of ferulic acid with pET-T5M and pET-opT5M. W, wild-type *E. coli*; T, tyrosine overproducing *E. coli*. Error bars indicate standard errors of the means. Different letters codes (a to c’) indicate significant differences (a, *P* = 2.06E-7; a’, *P* = 2.71E-12; b, *P* = 8.48E-4; b’, *P* = 4.3E-4; c, *P* = 1.67E-6; c’, *P* = 3.09E-7).

Under the same experimental conditions as before, strains overproducing tyrosine acquired a substantial capacity for 4-coumaric acid, caffeic acid and ferulic acid synthesis (Figure 4 and Table 2). As seen in Figure 4A and Table 2, tyrosine-overproducing *E. coli* (pAD-AG/*ΔtyrR*) with pET-TAL and pET-opTAL produced more than 974 ± 30 mg/L and 805 ± 41 mg/L of 4-coumaric acid, respectively. The production increased by 1650% and 559% over the parental strains, respectively. The similar production between native and codon-optimized TALs meant that the protein expression level was not a critical factor for the 4-coumaric acid production affecting the excessive tyrosine pool in our culture conditions.

**Table 2:** **Production of caffeic acid and ferulic acid by engineered tyrosine overproducing *E. coli *strains**

**Engineered tyrosine overproducing *E. coli *(pAD-AG/*ΔtyrR*)**	**4-Coumaric acid (mg/L)**	**Caffeic acid (mg/L)**	**Ferulic acid (mg/L)**
pET-TAL	**974 ± 30**^**a**^	*-*	*-*
pET-opTAL	**805 ± 41**^**a**^	*-*	*-*
pET-T5	307 ± 8	**150 ± 8**^**b**^	*-*
pET-opT5	511 ± 69	**40 ± 16**^**b**^	*-*
pET-T5M	113 ± 12	22 ± 3	**196 ± 26**^**c**^
pET-opT5M	150 ± 17	46 ± 7	**187 ± 12**^**c**^

Each batch cultivation was done at least in triplicate, and the standard deviations are shown. Different letter codes (a to c) indicate significant differences (a, *P* = 0.0216; b, *P* = 7.17E-07; c, *P* = 0.5176).

### Caffeic acid and ferulic acid production in the tyrosine overproducing strain

The titers of caffeic acid did not significantly improve (150 ± 8 mg/L and 40 ± 16 mg/L, respectively) in comparison to the 4-coumaric acid titers, despite 357% and 285% improvement over the parental strains (Table 2). As previously mentioned, the same artificial pathways (pET-T5 and pET-opT5) produced 42 mg/L and 14 mg/L of caffeic acid in the native tyrosine pathway of *E. coli* (Table 1). The titer of caffeic acid (150 ± 8 mg/L) with TAL (pET-T5) was higher than the titer (50 mg/L) of the pathway using 4-hydroxyphenylacetate 3-hydroxylase and TAL as recently reported by Lin et al. [15]. However, the caffeic acid titers with codon-optimized TAL and Sam5 co-expression reached only 4.9% of that of the 4-coumaric acid seen with the codon-optimized TAL expression alone (40 mg/L vs. 805 mg/L). In addition, the tyrosine overproducers containing pET-T5 and -opT5 also accumulated 307 mg/L and 511 mg/L of 4-coumaric acid, respectively (Table 2), and this result meant that the TAL enzymes operated normally, but that Sam5 did not convert 4-coumaric acid to caffeic acid well. Although caffeic acid concentrations were observed to be a somewhat low titer (40 ± 16 mg/L), the presence of any significant 4-coumaric acid accumulation (511 ± 69 mg/L) suggested that this discrepancy may be related to enhanced protein synthesis from the codon-optimized *tal* gene rather than 4-coumaric acid production and consumption.

On the other hand, ferulic acid synthesis levels in tyrosine-overproducing *E. coli* cells with pET-T5M and pET-opT5M were 196 ± 26 mg/L and 187 ± 12 mg/L, respectively. At the same time, the accumulation of 4-coumaric acid and caffeic acid also was identified at an expected amount (113 and 22 mg/L and 150 and 46 mg/L, respectively). As was the case for the previous result in wild-type *E. coli*, the titers of ferulic acid also show an increase over caffeic acid in the tyrosine-overproducing strain.

## Conclusions

We successfully established artificial biosynthetic pathways for precursor phenolic acids of phenylpropanoid and constructed *E. coli* strains for the *de novo* production of 4-coumaric, caffeic, and ferulic acids. The TALs from *S. espanaensis* and a codon-optimized synthetic gene expressed the native tyrosine pathway of *E. coli* and produced 59 ± 7 mg/L and 144 ± 14 mg/L of 4-coumaric acid from glucose sources (Figure 3). Further improvement of 4-coumaric acid production was accomplished by alleviating feedback inhibition and deregulating aromatic amino acids flux into tyrosine biosynthesis. Finally, the titers of 4-coumaric acid reached 974 ± 30 mg/L and 805 ± 41 mg/L in shake flasks after 36-hour cultivation (Table 2). We confirmed that codon optimization of the *tal* gene had a good effect on *de novo* production of 4-coumaric acid in a native tyrosine pathway, but the effect was offset by a sufficient tyrosine supply. More notably, this augmented precursor pool had a direct impact on 4-coumaric acid production, which increased 6.7-fold to yield a final titer of 974 ± 30 mg/L.

An artificial pathway leading from tyrosine to caffeic acid was then constructed and introduced into two kinds of *E. coli*. The caffeic acid levels increased to 150 mg/L and 40 mg/L in the tyrosine overproducer from just 42 mg/l and 14 mg/L in the native strain, respectively. The ferulic acid levels increased to 187 mg/L from 40 mg/L of caffeic acid in the tyrosine overproducer and to 73 mg/L from just 14 mg/L in the native strain.

As seen in Table 1, expressing all three genes that involved the codon-optimized *tal* gene led to a 2.6-fold increase in ferulic acid production comprising with native TAL expression in the parental strain (28 mg/L up to 73 mg/L). Similarly, the titer of 4-coumaric acid also increased 2.4-fold (59 mg/L up to 144 mg/L) in the same experimental conditions (Figure 3). However, in the tyrosine overproducing strain, the 4-coumaric acid (805 mg/L vs. 974 mg/L) and ferulic acid (187 mg/L vs. 196 mg/L) titers were similar to native and codon-optimized constructs. Therefore, we suggest that at least in our system the titer of an artificial biosynthetic pathway is more dependent on a higher metabolite (precursors and intermediates) concentration and/or flux than on each enzyme’s excess expression rate.

Finally, we achieved almost one gram per liter scale production of 4-coumaric acid. Maximum titers of 150 mg/L of caffeic acid and 196 mg/L of ferulic acid were achieved. Phenylpropanoic acids, such as 4-coumaric acid, caffeic acid, and ferulic acid, have great potential for pharmaceutical applications and food ingredients.

## Methods

### Bacterial strains, plasmids, and chemicals

*E. coli* DH5α and *E. coli* C41 (DE3) [23] were used for general DNA manipulation and expression of biosynthetic genes, respectively. A pCR®-TOPO vector (Invitrogen, Carlsbad, CA) was used for polymerase chain reaction (PCR) cloning. pET-28a(+) was purchased from Novagen (San Diego, CA). L-Tyrosine, 4-coumaric acid, caffeic acid, ferulic acid, and IPTG were purchased from Sigma-Aldrich (St. Louis, MO). Restriction enzymes (NEB), ExTaq (Takara Biochemicals Inc.), pfu (Solgent, Korea), an AccuPower® Ligation kit (Bioneer, Korea), and a Quick & Easy *E. coli* gene deletion kit (Gene Bridges, German) were used according to the instructions provided by the manufacturers.

### Codon optimization and synthesis of TAL, AroG^fbr^, and TyrA^fbr^

Codon optimization and synthesis of *tal* from *S. espanaensis* was performed with the GeneGPS™ program (DNA2.0). From here forward, synthetic genes/proteins are denoted by superscript “op” DNA sequences. Both AroG and TyrA feedback-inhibition resistance (fbr) derivatives were used to overcome the end-product inhibition of the respective enzymes. The AroG^fbr^ enzyme had an Asp-146-Asn substitution [24], and the TyrA^fbr^ enzyme comprised a Met-53-Ile in the chorismate mutase domain and Ala-354-Val substitution in the prephenate dehydrogenase domain [19]. Codon optimization and synthesis of the *aroG*^*fbr*^ and *tyrA*^*fbr*^ genes were also performed by DNA2.0.

### Heterologous pathway construction and assembly

A list of plasmids and strains used in this study can be found in Table 3. The plasmids were assembled by a serial stepwise cloning process as previously reported [10]. Briefly, the four genes (*tal*, *optal, sam5*, and *com*) were independently cloned into pET-28a(+). To construct an expression vector containing the three genes that were each under the control of an independent T7 promoter, we amplified the 1.76-kb DNA fragment containing the TAL coding region using pET-TAL as a template with primer TAL-N and CPac (the sequence is located downstream of the T7 terminator region of the pET vector and contains the designed *Pac*I site; Additional file 1: Table S2). Using the pET-Sam5 as a template, the 2.54-kb DNA fragment containing the *sam5* coding region was PCR-amplified with primer NPac (the sequence was located upstream of the T7 promoter region of the pET vector and contained the designed *Pac*I site; Additional file 1: Table S2) and CPac. The amplified fragments were digested with each restriction enzyme and cloned between *Pac*I- and *Hin*dIII-digested pET-28a(+) by a three-fragment ligation, resulting in pET-T5. To construct a pET-T5M vector, the 2.54-kb DNA fragment containing the *sam5* coding region was PCR-amplified with primer NPac (the sequence was located upstream of the T7 promoter region of the pET vector and contained the designed *Pac*I site; Additional file 1: Table S2) and CPac. In addition, the 1.88-kb DNA fragment containing the COM coding region was PCR-amplified using pET-COM as a template with primer NPac and COM-R. The amplified fragments were digested with each of the restriction enzymes and cloned between *Bam*HI- and *Hin*dIII-digested pET-28a(+) by a four-fragment ligation, resulting in pET-T5M. The pET-opTAL, pET-opT5, and pET-opT5M vectors were constructed the same way as the previous vectors, pET-TAL, pET-T5, and pET-T5M, respectively. Gene sequences and orientations were verified by sequencing after each round of cloning.

**Table 3:** Plasmids and strains used in this study

**Plasmid or strain**	**Relevant Characteristics**	**Source**
Plasmids		
pET-28a(+)	f1 ori, T7 promoter, Kan^R^	Novagen
pET-22b(+)	f1 ori, T7 promoter Amp^R^	Novagen
pET-TAL	pET-28a(+) carrying TAL from *Saccharothrix espanaensis*	Choi.et al. [10]
pET-opTAL	pET-28a(+) carrying codon-optimized *S. espanaensis* TAL	This study
pET-Sam5	pET-28a(+) carrying Sam5 from *S. espanaensis*	Choi.et al. [10]
pET-COM	pET-28a(+) carrying COM from *Arabidopsis thaliana*	Choi.et al. [10]
pET-T5	pET-28a(+) carrying *S. espanaensis* TAL and Sam5	This study
pET-opT5	pET-28a(+) carrying codon-optimized TAL and *S. espanaensis* Sam 5	This study
pET-T5M	pET-28a(+) carrying *S. espanaensis* TAL, *S. espanaensis* Sam5, and *A. thaliana* COM	Choi.et al. [10]
pET-opT5M	pET-28a(+) carrying codon-optimized TAL, *S. espanaensis* Sam5, and *A. thaliana* COM	This study
pACYCDuet-1	p15A ori, double T7 promoter, Cm^R^	Novagen
pET28-tyrA*	pET-28a(+) carrying codon-optimized *tyr*A^fbr^	This study
pET22-aroG*	pET-22b(+) carrying codon-optimized *aro*G^fbr^	This study
pAD-AG	pACYDuet-1 carrying codon-optimized *tyr*A^fbr^ and *aro*G^fbr^	This study
Strains		
*E. coli* DH5a	cloning host	Invitrogen
*E. coli* C41(DE3)	derivative strain of *E. coli* BL21(DE3)	Miroux B & Walker JE [23]
*ΔtyrR*	*tryR* gene in-frame deletion mutant of *E. coli* C41(DE3)	This study
pAD-AG/Δ*tyrR*	*E. coli* C41(DE3) Δ*tryR* :: pAD-AG; tyrosine overproducing strain	This study

### In-frame deletion of the *tyrR* gene

The genetic design of the L-tyrosine overproducing strain was followed as previously described by Lütke-Eversloh and Stephanopoulos [18]. The in-frame deletion of *tyrR*, a repressor gene of aromatic amino acid biosynthesis, was constructed by RED/ET recombination with a Quick & Easy *E. coli* Gene deletion kit (Gene Bridges) using the manufacturer’s protocols. The PCR recombination product was generated using the FRT-PGK-gb2-neo-FRT fragment as a template for the FRT-flanked kanamycin cassette and the following primers: tyr-Rf (5^′^-GTCATATCAT CATATTAAT TGTTCTTTTT TCAGGTGAAG GTTCCCATGC GTAATTAACC CTCACTAAAG GGCG-3^′^) and tyr-Rr (5^′^-ATCAGGCATA TTCGCGCTTA CTCTTCGTTC TTCTTCTGAC TCAGACCATA TAATACGACT CACTATAGGG CTC-3^′^). The kanamycin selection marker was removed from the chromosome by transforming the cells with an FLP recombinase expression plasmid, 706-FLP (Gene Bridges). The clones growing on the kanamycin plate still contained the selection marker cassette, while all other clones containing in-frame deletion lost the selection marker. The in-frame deletion mutant (Δ*tyrR*) was verified through PCR using the following primers: tyr-v1 (5^′^- AACCT CGCCT CGGGG ATTTC −3^′^) and tyr-v2 (5^′^- AGCGC GTGCC GTTGT GGTTA −3^′^). The PCR product was sequenced and verified.

### Construction of L-tyrosine overproducing strain

The L-tyrosine over-producing *E. coli* strain was achieved by overexpression of *aroG*^*fbr*^ and *tyrA*^*fbr*^ genes in the in-frame deletion Δ*tyrR* mutant. To elevate the expression levels of the *aroG*^*fbr*^ and *tyrA*^*fbr*^ genes, the RBS and the T7 promoter were positioned in front of both genes. A synthetic *aroG*^*fbr*^ DNA fragment (from DNA2.0) was digested with restriction enzymes *Nde*I- and *Hin*dIII, and the appropriate fragments were ligated into pET-22b(+) to form pET22-aroG* (Table 3). The pET28-tyrA*, which contained a codon-optimized 1.1-kb *tyrA*^*fbr*^ coding region, was digested with restriction enzymes and cloned between *Nco*I- and *Hin*dIII restriction enzyme sites of MCS1 of pACYCDuet-1, resulting in pAD-tyrA*. Then, the 1.1-kb DNA fragment containing the *aroG*^*fbr*^ coding region of the pET22-aroG* was digested with restriction enzymes *Nde*I and *Xho*I, including the *Hin*dIII site of the stop codon, and cloned between the *Nde*I- and *Xho*I restriction enzyme sites of MCS2 of pAD-tyrA*, resulting in pAD-AG (Table 3). The L-tyrosine over-producing *E. coli* strain (pAD-AG/Δ*tyrR*) was achieved by a Δ*tyrR* mutant carrying pAD-AG.

### Culture conditions for production

Recombinant *E. coli* C41 (DE3) strains harboring plasmids were precultured overnight at 37°C in a Luria–Bertani (LB) medium containing 50 μg/mL kanamycin. The overnight culture was inoculated (1.5%) into a fresh LB medium supplemented with the same concentration of kanamycin. The culture was grown at 37°C to an optical density of 600 nm (OD_600_) of 0.6. IPTG was added to the final concentration of 1 mM, and the culture was incubated for 5 hours. The cells were harvested by centrifugation, suspended, and incubated at 26°C for 36 hours in a modified M9 medium (M9 medium supplemented with 15 g/L glucose, 25 g/L CaCO_3_, appropriate antibiotics (50 μg/mL kanamycin for maintenance of pET-derived plasmids and 34 μg/mL chloramphenicol for pACYC-derived plasmid (pAD-AG)), and 1 mM IPTG) [10,25]. The samples were collected after 36 hours and analyzed by HPLC.

### Detection and quantification of the products

For the quantification of L-tyrosine, 4-coumaric acid, caffeic acid, and ferulic acid, 1 mL of cell-free culture supernatants were filtered through 0.2 μm Cellulose membrane syringe filters (Sartorius) and used for HPLC analysis with a Dionex Separations module connected with a Photodiode Array detector (Dionex) set. The compounds were separated by elution with an acetonitrile-water gradient (water containing 0.05% trifluoroacetic acid (TFA)). The L-tyrosine was separated on a YMC C18 column (150 × 4.6 mm, 4 μm). The following gradient was used at a flow rate of 1 mL/min: 5% to 80% acetonitrile for 25 min, 80% to 100% acetonitrile for 3 min, 100% acetonitrile for 3 min, 100% to 5% acetonitrile for 3 min, and 5% acetonitrile for an additional 3 min. The 4-coumaric acid was separated on a YMC C18 column. The following gradient was used at a flow rate of 1 mL/min: 20% to 60% acetonitrile for 25 min, 60% to 100% acetonitrile for 1 min, 100% acetonitrile for 5 min, 100% to 20% acetonitrile for 2 min, and 20% acetonitrile for an additional 5 min. The caffeic acid was separated on a YMC C18 column. The following gradient was used at a flow rate of 1 mL/min: 15% acetonitrile for 5 min, 15% to 60% acetonitrile for 15 min, 60% to 100% acetonitrile for 5 min, 100% acetonitrile for 5 min, 100% to 15% acetonitrile for 2 min, and 15% acetonitrile for an additional 5 min. The ferulic acid was separated on a SunFire™ C18 column (250 × 4.6 mm, 5 μm; Waters, USA). The following gradient was used at a flow rate of 1 mL/min: 20% to 25% acetonitrile for 15 min, 25% to 100% acetonitrile for 5 min, 100% acetonitrile for 5 min, 100% to 20% acetonitrile for 5 min, and 20% acetonitrile for an additional 5 min. Quantification for the four above-mentioned compounds was based on the peak areas of absorbance at 250 nm (L-tyrosine), 300 nm (4-coumaric acid) and 320 nm (caffeic acid and ferulic acid). The concentrations were determined through the use of the corresponding chemical standards (Sigma).

The data shown in this study were generated from triplicate independent experiments. The titers of each production were compared by single-factor ANOVA (P < 0.05) using the single-factor ANOVA tool. The Tukey honestly significant difference (HSD) test was used to determine the significance of differences between group means. The data were analyzed using Microsoft Office Excel 2007 and IBM SPSS Statistics.

## Competing interests

The authors declare that they have no competing interests.

## Authors’ contributions

SK and OC performed the experiments and wrote the manuscript. JL co-performed the experiments on the metabolite analysis. BH and TU contributed general advice, particularly on the metabolite analysis and resource support. YH designed all the experiments and wrote the manuscript. All authors read and approved the final manuscript.

## Supplementary Material

Additional file 1 **Figure S1.** Comparison of the TAL protein expression of pET-opTAL and pET-TAL in *E. coli*. Lane 1: *E. coli* transformation containing pET-opTAL expression vector lysate after IPTG induction; Lane 2: lysate before induction; Lane 3: *E. coli* transformation containing pET-TAL expression vector lysate after IPTG induction; Lane 4: lysate before induction. **Figure S2.** SDS-PAGE analysis of co-expression of TAL and Sam5 enzymes in *E. coli*. Lane 1: *E. coli* transformation containing pET-opT5 expression vector lysate before IPTG induction; Lane 2: lysate after induction; Lane 3: *E. coli* transformation containing pET-T5 expression vector lysate before IPTG induction; Lane 4: lysate after induction. Sam5 protein is 59 kDa, TAL protein from pET-opT5 expression vector is 56 kDa, TAL protein from pET-T5 expression vector is 57.4 kDa. The difference in the TAL proteins comes from the length of the His-tagged peptide sequences. **Table S1.** The results of the Tukey test for the data from Figure 3. **Table S2.** Oligo nucleotide primers used in this study. Click here for file
