# Supplementary material for: Artificial biosynthesis of phenylpropanoic acids in a tyrosine overproducing Escherichia coli strain
Source: Microb Cell Fact. 2012 Dec 3;11:153. doi: 10.1186/1475-2859-11-153 (PMC3554431; doi:10.1186/1475-2859-11-153)
Supplement: Additional file 1 — Figure S1. Comparison of the TAL protein expression of pET-opTAL and pET-TAL in E. coli. Lane 1: E. coli transformation containing pET-opTAL expression vector lysate after IPTG induction; Lane 2: lysate before induction; Lane 3: E. coli transformation containing pET-TAL expression vector lysate after IPTG induction; Lane 4: lysate before induction. Figure S2. SDS-PAGE analysis of co-expression of TAL and Sam5 enzymes in E. coli. Lane 1: E. coli transformation containing pET-opT5 expression vector lysate before IPTG induction; Lane 2: lysate after induction; Lane 3: E. coli transformation containing pET-T5 expression vector lysate before IPTG induction; Lane 4: lysate after induction. Sam5 protein is 59 kDa, TAL protein from pET-opT5 expression vector is 56 kDa, TAL protein from pET-T5 expression vector is 57.4 kDa. The difference in the TAL proteins comes from the length of the His-tagged peptide sequences. Table S1. The results of the Tukey test for the data from Figure 3. Table S2. Oligo nucleotide primers used in this study. [file 1475-2859-11-153-S1.pptx]

## Slide 1
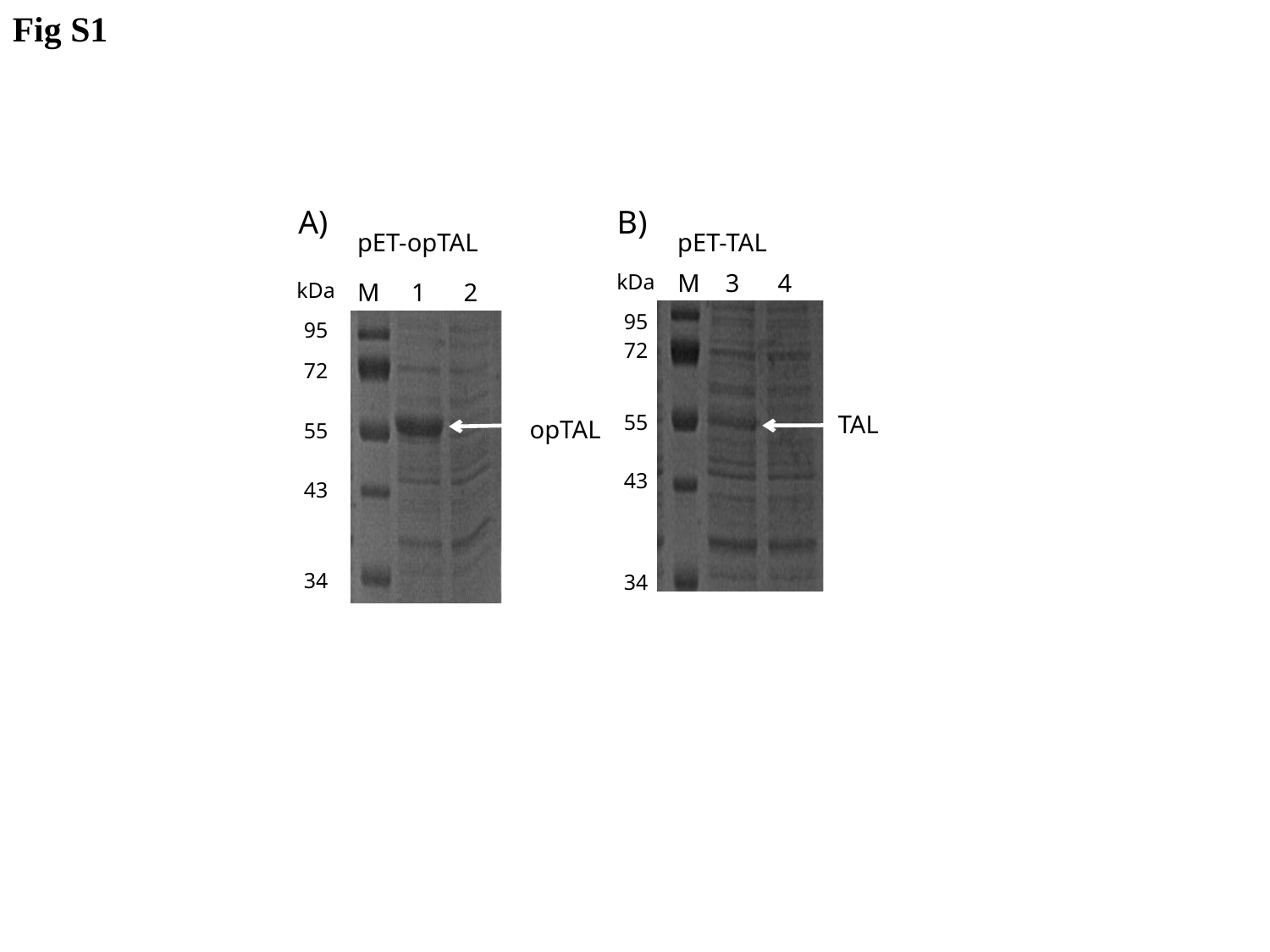

Fig S1
A)
B)
pET-opTAL
pET-TAL
M 3 4
kDa
95
72
55
43
34
kDa
95
72
55
43
34
M 1 2
TAL
opTAL
*

## Slide 2
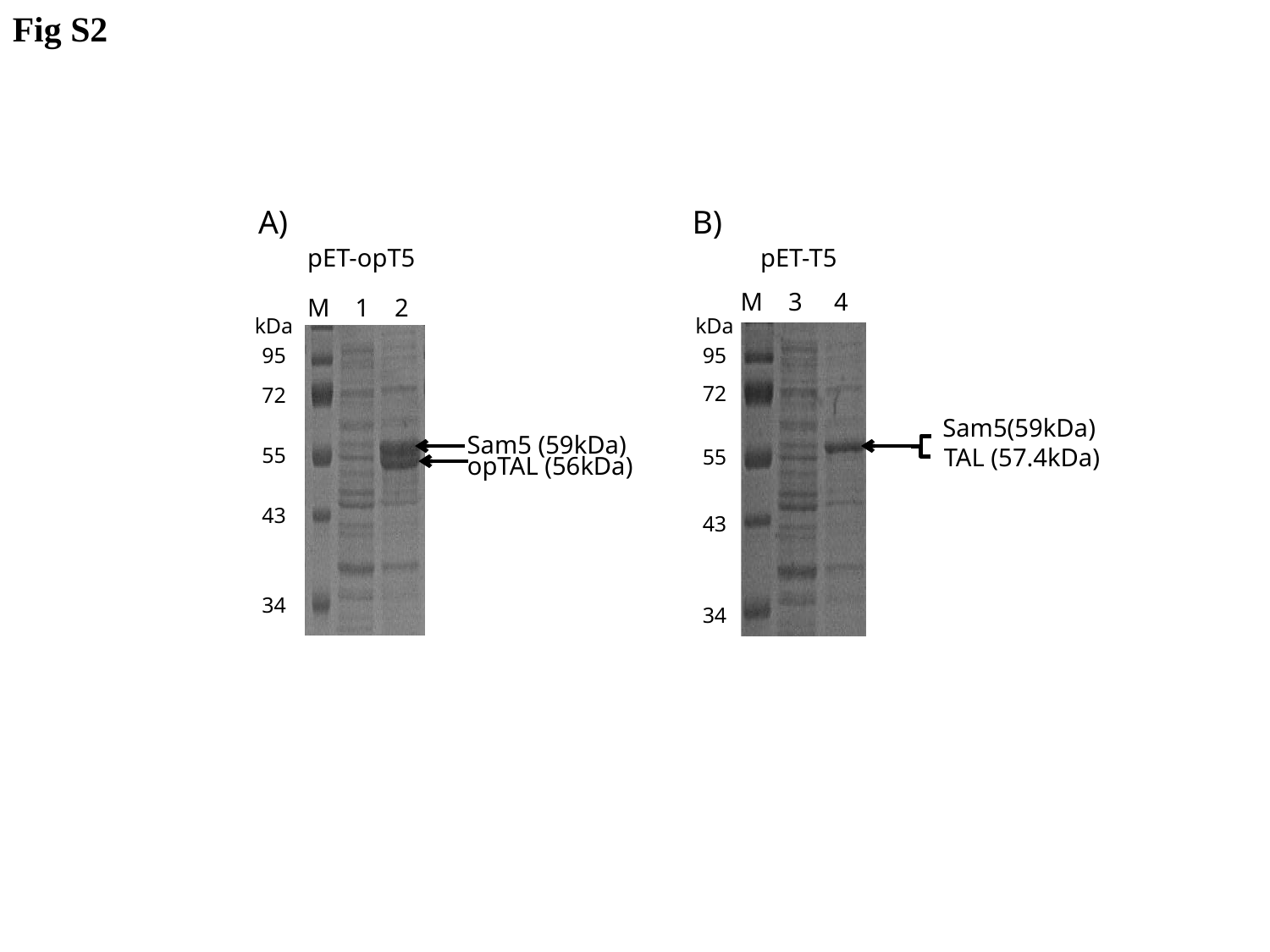

Fig S2
A)
pET-opT5
M 1 2
kDa
95
72
Sam5 (59kDa)
55
opTAL (56kDa)
43
34
B)
pET-T5
M 3 4
kDa
95
72
Sam5(59kDa)
TAL (57.4kDa)
55
43
34

## Slide 3
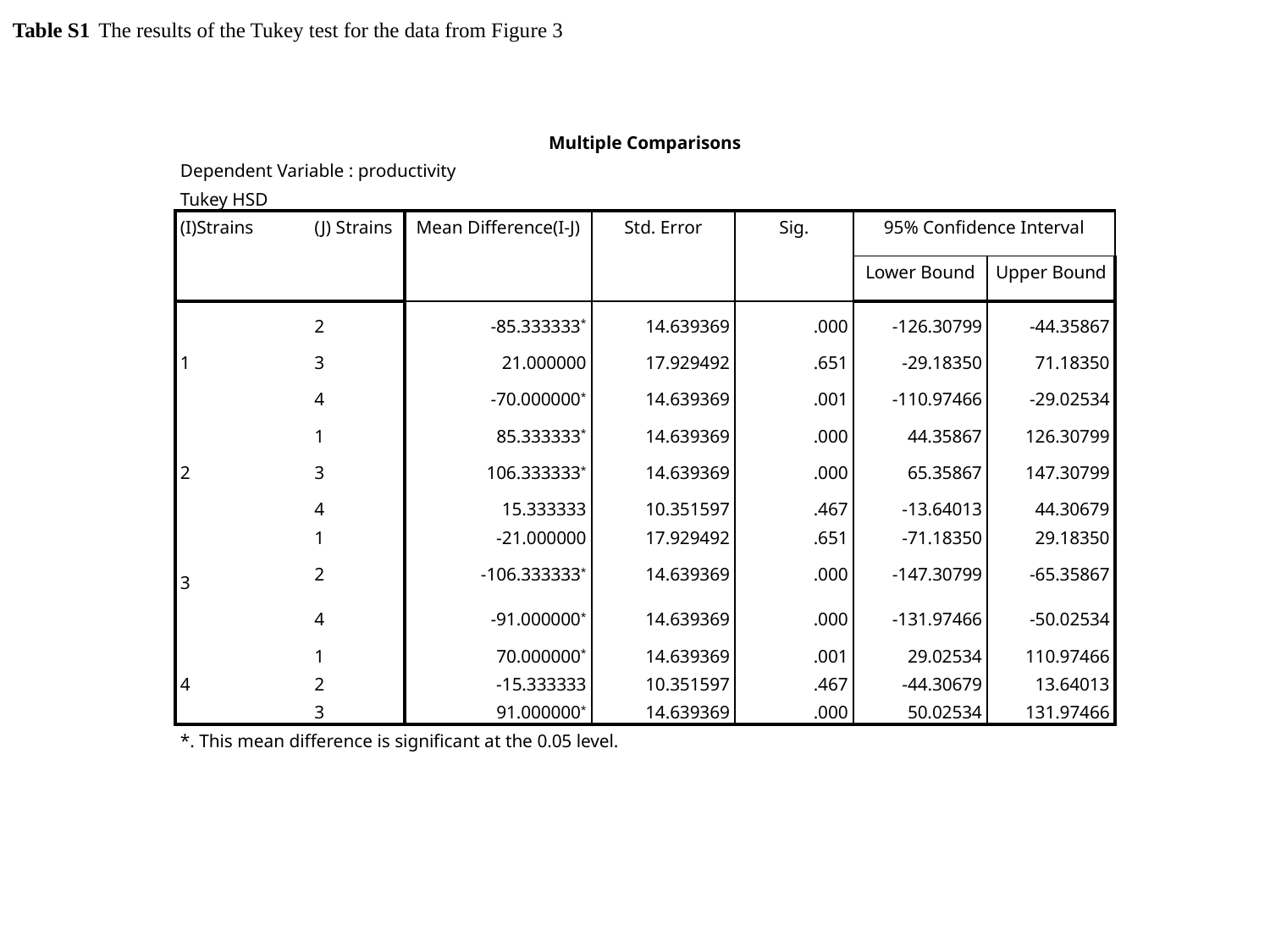

Table S1 The results of the Tukey test for the data from Figure 3
| Multiple Comparisons Dependent Variable : productivity | | | | | | |
| --- | --- | --- | --- | --- | --- | --- |
| Tukey HSD | | | | | | |
| (I)Strains | (J) Strains | Mean Difference(I-J) | Std. Error | Sig. | 95% Confidence Interval | |
| | | | | | Lower Bound | Upper Bound |
| 1 | 2 | -85.333333\* | 14.639369 | .000 | -126.30799 | -44.35867 |
| | 3 | 21.000000 | 17.929492 | .651 | -29.18350 | 71.18350 |
| | 4 | -70.000000\* | 14.639369 | .001 | -110.97466 | -29.02534 |
| 2 | 1 | 85.333333\* | 14.639369 | .000 | 44.35867 | 126.30799 |
| | 3 | 106.333333\* | 14.639369 | .000 | 65.35867 | 147.30799 |
| | 4 | 15.333333 | 10.351597 | .467 | -13.64013 | 44.30679 |
| 3 | 1 | -21.000000 | 17.929492 | .651 | -71.18350 | 29.18350 |
| | 2 | -106.333333\* | 14.639369 | .000 | -147.30799 | -65.35867 |
| | 4 | -91.000000\* | 14.639369 | .000 | -131.97466 | -50.02534 |
| 4 | 1 | 70.000000\* | 14.639369 | .001 | 29.02534 | 110.97466 |
| | 2 | -15.333333 | 10.351597 | .467 | -44.30679 | 13.64013 |
| | 3 | 91.000000\* | 14.639369 | .000 | 50.02534 | 131.97466 |
| \*. This mean difference is significant at the 0.05 level. | | | | | | |

## Slide 4
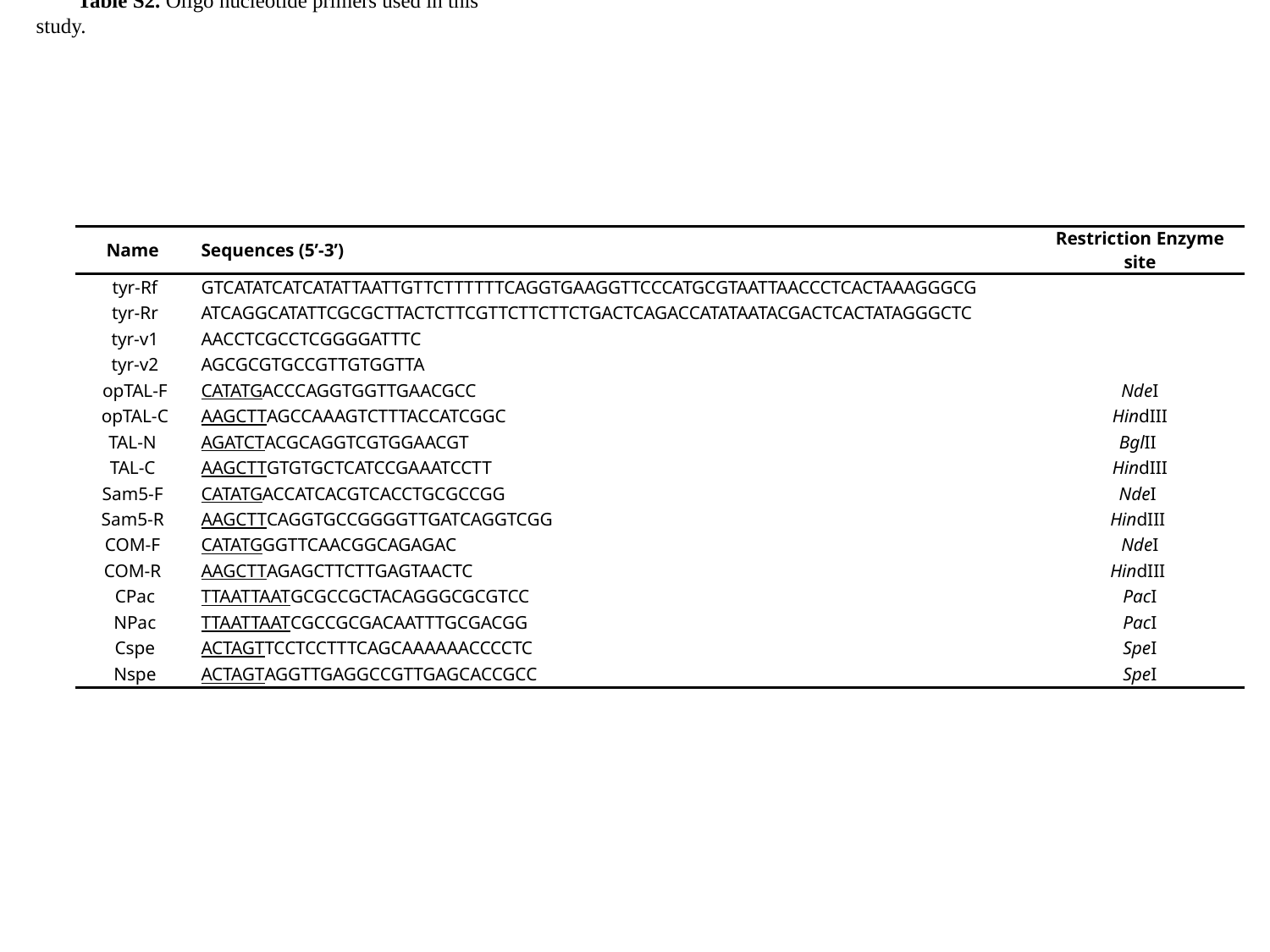

Table S2. Oligo nucleotide primers used in this study.
| Name | Sequences (5’-3’) | Restriction Enzyme site |
| --- | --- | --- |
| tyr-Rf | GTCATATCATCATATTAATTGTTCTTTTTTCAGGTGAAGGTTCCCATGCGTAATTAACCCTCACTAAAGGGCG | |
| tyr-Rr | ATCAGGCATATTCGCGCTTACTCTTCGTTCTTCTTCTGACTCAGACCATATAATACGACTCACTATAGGGCTC | |
| tyr-v1 | AACCTCGCCTCGGGGATTTC | |
| tyr-v2 | AGCGCGTGCCGTTGTGGTTA | |
| opTAL-F | CATATGACCCAGGTGGTTGAACGCC | NdeI |
| opTAL-C | AAGCTTAGCCAAAGTCTTTACCATCGGC | HindIII |
| TAL-N | AGATCTACGCAGGTCGTGGAACGT | BglII |
| TAL-C | AAGCTTGTGTGCTCATCCGAAATCCTT | HindIII |
| Sam5-F | CATATGACCATCACGTCACCTGCGCCGG | NdeI |
| Sam5-R | AAGCTTCAGGTGCCGGGGTTGATCAGGTCGG | HindIII |
| COM-F | CATATGGGTTCAACGGCAGAGAC | NdeI |
| COM-R | AAGCTTAGAGCTTCTTGAGTAACTC | HindIII |
| CPac | TTAATTAATGCGCCGCTACAGGGCGCGTCC | PacI |
| NPac | TTAATTAATCGCCGCGACAATTTGCGACGG | PacI |
| Cspe | ACTAGTTCCTCCTTTCAGCAAAAAACCCCTC | SpeI |
| Nspe | ACTAGTAGGTTGAGGCCGTTGAGCACCGCC | SpeI |
